# Fatty Acid β-Oxidation Is Essential in Leptin-Mediated Oocytes Maturation of Yellow Catfish *Pelteobagrus fulvidraco*

**DOI:** 10.3390/ijms19051457

**Published:** 2018-05-14

**Authors:** Yu-Feng Song, Xiao-Ying Tan, Ya-Xiong Pan, Li-Han Zhang, Qi-Liang Chen

**Affiliations:** 1 Key Laboratory of Freshwater Animal Breeding, Ministry of Agriculture of China, Fishery College, Huazhong Agricultural University, Wuhan 430070, China; syf880310@mail.hzau.edn.cn (Y.-F.S.); biopyx@webmail.hzau.edu.cn (Y.-X.P.); zhlh8175@126.com (L.-H.Z.); xncql@126.com (Q.-L.C.); 2 Collaborative Innovation Center for Efficient and Health Production of Fisheries, Hunan University of Arts and Science, Changde 415000, China

**Keywords:** *Pelteobagrus fulvidraco*, β-oxidation, oocytes maturation, leptin, JAK-STAT pathway

## Abstract

Although several studies have been conducted to study leptin function, information is very scarce on the molecular mechanism of leptin in fatty acid β-oxidation and oocytes maturation in fish. In this study, we investigated the potential role of fatty acid β-oxidation in leptin-mediated oocytes maturation in *Pelteobagrus fulvidraco*. Exp. 1 investigated the transcriptomic profiles of ovary and the differential expression of genes involved in β-oxidation and oocytes maturation following rt-hLEP injection; rt-hLEP injection was associated with significant changes in the expression of genes, including twenty-five up-regulated genes (*CPT1*, *Acsl*, *Acadl*, *Acadm*, *Hadhb*, *Echsl*, *Hsd17b4*, *Acca*, *PPARα*, *CYP8B1*, *ACOX1*, *ACBP*, *MAPK*, *RINGO*, *Cdc2*, *MEK1*, *IGF-1R*, *APC/C, Cdk2*, *GnRHR, STAG3*, *SMC1*, *FSHβ* and *C-Myc*) and ten down-regulated gene (*PPARγ*, *FATCD36*, *UBC*, *PDK1*, *Acads*, *Raf*, *Fizzy*, *C3H-4*, *Raf* and *PKC*), involved in fatty acid β-oxidation and oocytes maturation. In Exp. 2, rt-hLEP and specific inhibitors AG490 (JAK-STAT inhibitor) were used to explore whether leptin induced oocytes maturation, and found that leptin incubation increased the diameters of oocytes and percentage of germinal vesicle breakdown (GVBD)-MII oocytes, up-regulated mRNA levels of genes involved in oocytes maturation and that leptin-induced oocyte maturation was related to activation of JAK-STAT pathway. In Exp. 3, primary oocytes of *P. fulvidraco* were treated with (R)-(+)-etomoxir (an inhibitor of β-oxidation) or l-carnitine (an enhancer of β-oxidation) for 48 h under rt-hLEP incubation. Exp. 3 indicated that the inhibition of fatty acid β-oxidation resulted in the down-regulation of gene expression involved in oocytes maturation, and repressed the leptin-induced up-regulation of these gene expression. Activation of fatty acid β-oxidation improved the maturation rate and mean diameter of oocytes, and up-regulated gene expression involved in oocytes maturation. Leptin is one of the main factors that links fatty acid β-oxidation with oocyte maturation; β-oxidation is essential for leptin-mediated oocyte maturation in fish.

## 1. Introduction

In fish, the oocyte undergoes meiotic resumption after completion of vitellogenic process. This event, termed as meiotic maturation or oocytes maturation, is an important step in oogenesis (i.e., the process resulting in the release of fertilizable oocytes at ovulation). The rapid and tightly synchronised events, including proliferation of granulosa cell, production of cumulus cell matrix and segregation of chromosome, are energy-consuming processes and require adequate ATP generation from cellular energy stores [1,2].

Fatty acids are widely known as potential metabolic substrates for the oocytes, providing an efficient source of energy upon requirement [2], mainly by fatty acid β-oxidation for the ATP production in mitochondria. Crucial roles of fatty acids in promoting embryo development in mammals have been clearly indicated and comprehensively reviewed [3]. However, accumulating evidence demonstrates that the metabolism of fatty acids by β-oxidation in the cumulus–oocyte complex (COC) before fertilisation has also impacts on subsequent oocyte developmental potentials [2]. Oocytes and cumulus cells are well known to contain lipid droplets, but how these are utilised during oocytes maturation remains largely unknown. In mammals, the fatty acid β-oxidation was first implied by Hillman and Flynn [4] within ovulated mouse oocytes, in which incorporation of ^14^C-palmitic acid into oocytes was found. Studies using pharmacological inhibitors have determined the essential role of fatty acid β-oxidation in both oocyte nuclear maturation and developmental competence [5,6]. In addition, up-regulation of fatty acid β-oxidation in COCs has also been used to demonstrate the crucial role of this metabolic pathway for developmental competence in vitro [7,8]. However, all of these studies only involved the changes of very limited genes’ mRNA levels related to the fatty acid β-oxidation during oocyte maturation [7,9]. Furthermore, in fish, up to date, very few studies are conducted to elucidate the potential role of the fatty acid β-oxidation in oocytes maturation. Compared to mammals, most of fish belong to oviparous animals. There are significant differences in ovary development and oocyte maturations between mammals and fish. Therefore, it remains cautious when extrapolating the results in mammals to fish.

Leptin, a product of the obese gene [10], acts on the hypothalamus to regulate energy metabolism [11]. Previous studies in mammals indicated that leptin stimulated the oxidation of fatty acids, and prevents the accumulation of lipids [12]. Due to the long-known relationship between developmental competence and nutritional status, recent studies have focused on the role of leptin in regulation of developmental competence [13]. The expression of leptin and its receptors has been demonstrated in cumulus cells, oocytes, and ovary from mammals to fish [14,15], implying that leptin can regulate reproductive activities at various levels of the hypothalamic-pituitary-gonadal axis. In mammals, studies have also indicated a potential direct role for leptin in regulation of ovarian function and oocytes maturation [16,17,18]. Studies pointed out that leptin stimulated the hypothalamic–pituitary–gonadal axis from the hypothalamus by promoting the release of gonadotropin releasing hormone (GnRH), and follicle-stimulating hormone (FSH) and luteinizing hormone (LH) from the pituitary resulting in a cascade of hormonal responses that promote reproduction [19,20]. In fishes, several studies also reported that leptin could stimulate the reproductive axis and increased the release of FSH and LH [21,22]. On the other hand, mounting evidence indicated that leptin exerted its biological actions primarily through the janus kinase/signal transducer and activator of transcription (JAK-STAT) signaling pathway [23]. Thus, JAK-STAT pathway may mediate leptin-induced oocytes maturation. Additionally, given the critical role of leptin in regulation of fatty acid β-oxidation, it is also imperative to investigate the potential role of fatty acid β-oxidation in the leptin-mediated oocytes maturation.

Yellow catfish *Pelteobagrus fulvidraco*, an omnivorous freshwater fish, is regarded as a potential model for studying the link between fatty acid metabolism and ovary development in fish. Recently, in our laboratory, we cloned and characterized cDNA sequences of leptin and leptin receptor in yellow catfish, and found the predominant mRNA expression of leptin and its receptor in ovary, inferring the direct role of leptin in ovary [14]. Furthermore, in our laboratory, Zhang et al. [13] pointed out that recombinant human leptin (rt-hLEP) incubation enhanced the activity and expression of carnitine palmitoyltransferase (CPT-1, a rate-limiting enzyme of fatty acid oxidation) in ovarian follicle cells from *P. fulvidraco*, indicating that leptin had a direct effect on fatty acid β-oxidation. However, due to the lack of genomic resources such as genome and transcriptome sequences of yellow catfish, these earlier studies only provided limited information. Clearly, a global understanding of the ovarian transcriptomic profiling of *P. fulvidraco* could underpin efforts to comprehensively understand the underlying molecular processes involved in leptin-mediated fatty acid β-oxidation and oocytes maturation. Therefore, in this study, the ovarian transcriptome information of *P. fulvidraco* by RNA-seq technology was determined, and also the differential expression of genes involved in fatty acid β-oxidation and oocytes maturation following rt-hLEP administration were investigated. Then, we explored whether leptin induced oocytes maturation and its relationship with JAK-STAT pathway. Furthermore, through inhibition and up-regulation of the fatty acid β-oxidation pathway, we investigated the potential effect of fatty acid β-oxidation on leptin-mediated oocytes maturation competence. To our knowledge, our study, for the first time, provides evidence that leptin is a very important factor that links fatty acid β-oxidation with oocytes maturation in fish; leptin promotes oocytes maturation competence via activation of JAK-STAT pathway; fatty acid β-oxidation is essential for leptin-mediated oocyte maturation competence, and the up-regulation of fatty acid β-oxidation improves leptin-mediated oocytes maturation in fish.

## 2. Results

### 2.1. Ovary Transcriptome of P. fulvidraco Generated by RNA-Seq

To obtain an overview of gene expression profile in ovary of *P. fulvidraco*, cDNA libraries were constructed from the ovaries of control and rt-hLEP-injected fish and were subjected to Illumina sequencing platform. A total of 50,181,922 and 41,408,858 clean reads with 5,018,192,200 and 4,140,885,800 nucleotides (nt) were remained, respectively (Table 1). The Q20, GC content and unknown bases (N) were 97.19%, 48.55% and 0.00%, respectively, for the control sample, and 97.95%, 48.03% and 0.00%, respectively, for the rt-hLEP-injected sample (Table 1). These reads were assembled, resulting in 76,153, and 75,384 contigs, respectively, yielding 36,149 unigenes (All-Unigene), with average length of 1382 bp (N50 = 2440 bp. N50 = median length of all non-redundant sequences) (Table 2). The length distribution of All-Unigene was shown in Supplementary Figure S1. The sequencing data in this study have been deposited in the Short Read Archive (SRA) at the National Center for Biotechnology Information (NCBI) (accession number: 352 SRX1080987).

**Figure 1:**
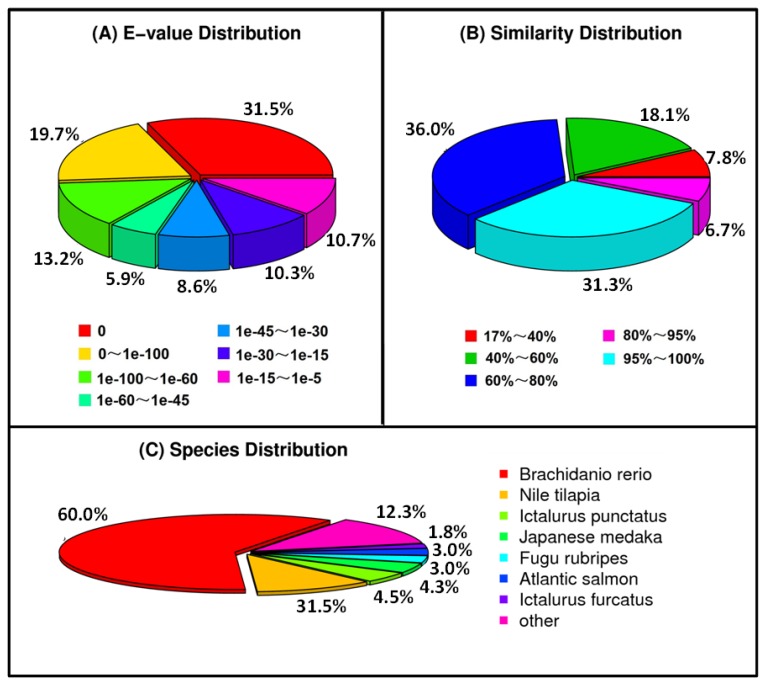
Homology analysis of *P. fulvidraco* transcriptome. All distinct gene sequences that had BLAST annotations within the NR database with a cut-off *e*-value ≤10^−5^ were analysed for *e*-value distribution (**A**), similarity distribution (**B**), and species distribution (**C**).

A total of 24,275 (67.15%) and 22,768 (62.98%) unigenes were unambiguous alignments to the reference when BLASTx against NR and Swiss-Prot database, respectively, while BLASTn against NT database returned 23,751 (65.70%) hits. The e-value distribution of the top hits showed that 51.2% of the mapped sequences have strong homology (<1e-100) (Figure 1A). The similarity distribution showed that 38.0% of the sequences had a similarity >80% (Figure 1B). For species distribution, 60.0% of the matched unigenes showed similarities with *Brachidanio rerio*, followed by *Nile tilapia* (31.5%), *Ictalurus punctatus* (4.5%), and *Japanese medaka* (4.3%) (Figure 1C).

**Figure 2:**
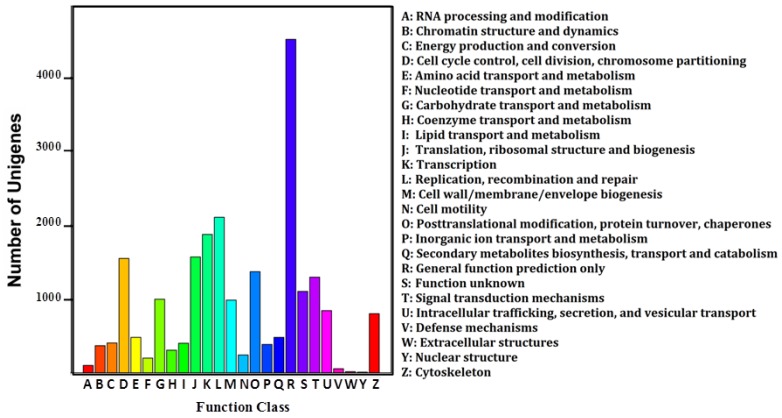
COG function classification of All-Unigene. The horizontal coordinates are function classes of COG, and the vertical coordinates are numbers of unigenes in one class. The notation on the right is the full name of the functions in *X* axis.

A total of 16,835 (46.57%) GO terms were obtained (Supplementary Table S2), with 53.20% for biological processes, 34.04% for cellular components and 12.76% for molecular functions. The three main GO categories were classified into 58 subcategories (Supplementary Figure S2). The COG assignments were performed to predict and classify possible functions of unigenes. A total of 9852 (27.25%) sequences were functionally classified into 25 COG categories (Supplementary Table S2; Figure S2). Among these categories, most enriched terms were “General function of prediction only”, followed by “Replication, recombination and repair”, and “Transcription” (Figure 2). Besides the GO and COG analysis, Kyoto Encyclopedia of Genes and Genomes (KEGG) pathway parsing was also carried out. A total of 18,950 (52.42%) unigenes were mapped into 184 signaling pathways (Supplementary Table S2), including JAK-STAT signaling pathway, fatty acid β-oxidation pathway, progesterone-mediated oocyte maturation pathway, and oocyte meiosis signaling pathway.

### 2.2. Leptin Is a Very Important Factor That Links Fatty Acid β-Oxidation with Oocytes Maturation

**Figure 3:**
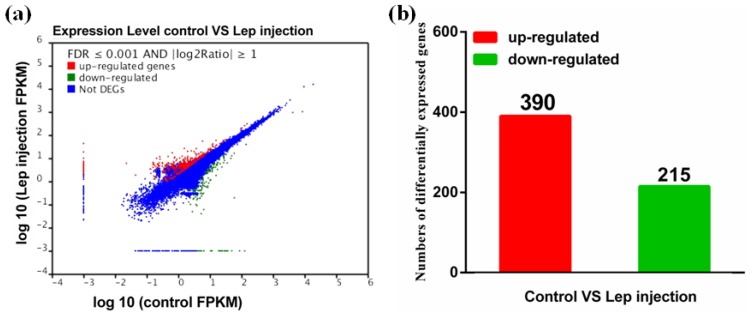
(**a**) Scatter plots showing gene expression profiles in the ovary of *P. fulvidraco* from the control and rt-hLEP-injected groups; (**b**) histogram showing numbers of differentially expressed genes in the ovary of *P. fulvidraco* from the control and rt-hLEP-injected groups. Differentially expressed genes are indicated in red (up-regulation) and green (down-regulation). Blue indicates genes that were not differentially expressed.

**Figure 4:**
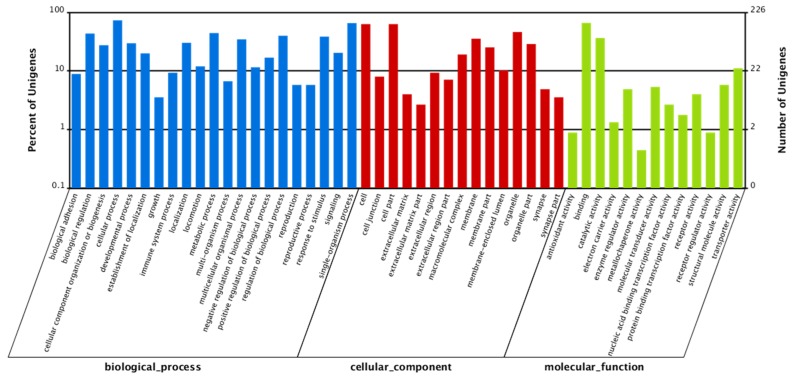
GO classification of differentially expressed unigenes. Unigenes were assigned to three main categories: biological process, cellular components, and molecular function. Values are displayed for each term as the percentage of the total number of genes as well as the number of genes.

In order to investigate the role and molecular mechanism of leptin underlying fatty acid β-oxidation and oocytes maturation, we analysed all relevant differentially expressed genes in transcriptome. There were a total of 605 genes identified as differentially expressed genes among two treatments, of which 390 were up-regulated and 215 were down-regulated in the ovary from the control compared with those from rt-hLEP-injected fish (Figure 3). For the GO analysis, 178, 193 and 184 differentially expressed genes were grouped in cellular component, molecular function and biological process categories, respectively (Figure 4).

To characterize the functional consequences of gene expression changes associated with rt-hLEP injection, pathway analysis was performed based on the KEGG database. Of the 605 differentially expressed genes, 309 had a specific KEGG pathway annotation, indicating rt-hLEP injection posed significant effects on different biological pathways, such as signal transduction involved in the processes of leptin exerting its biological actions, fatty acid metabolism and oocytes maturation.

#### 2.2.1. Differentially Expressed Genes Involved in Signal Transduction

**Figure 5:**
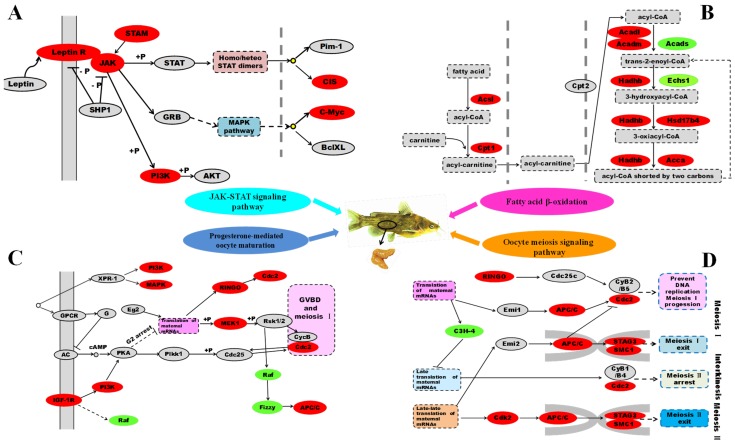
Leptin is a very important factor that links fatty acid β-oxidation with oocytes maturation. Differentially expressed genes between the control and rt-hLEP-injected groups from transcriptome and DGE analysis. The most important pathways related to fatty acid β-oxidation and oocytes maturation included JAK-STAT signalling pathway (**A**), fatty acid β-oxidation (**B**), progesterone-mediated oocyte maturation (**C**), oocyte meiosis signalling pathway (**D**). The map was drawn by ourselves based on KEGG databases and on studies by Kobayashi et al. [24] and Collins et al. [25] Genes with green or red background indicated the mRNA expression levels of rt-hLEP-injected fish were significantly lower or higher than those in the control, respectively (FDR ≤ 0.001, the absolute value of log2[Ratio] ≥1). Solid arrows means direct or known interaction. Dotted arrows means indirect link or unknown interaction.

To determine the involvement of signal transduction pathway in leptin influencing fatty acid β-oxidation and oocytes maturation, we analyzed and identified several signal transduction related pathways, including JAK-STAT signaling pathway, mitogen-activated protein kinase (MAPK) signaling pathway, and AMPK signaling pathway (Table 3). The vast majority of genes involved in these significant pathways were up-regulated following rt-hLEP injection, such as *Leptin R*, *JAK*, *PI3K*, *MEKK*, *IGF-1R* and *AMPK* (Table 3; Figure 5A).

#### 2.2.2. Differentially Expressed Genes Involved in Fatty Acid β-Oxidation

In order to confirm the effects of rt-hLEP injection on fatty acid β-oxidation, analysis of differentially expressed genes involved in β-oxidation revealed that fatty acid β-oxidation pathway was enriched, including eight up-regulated genes (*CPT1*, *Acsl*, *Acadl*, *Acadm*, *Hadhb*, *Echsl*, *Hsd17b4*, and *Acca*) and one down-regulated gene (*Acads*) (Table 3; Figure 5B). Moreover, peroxisome proliferator-activated receptor (PPAR) signaling pathway was enriched as well, with five up-regulated genes (*CPT1*, *PPARα*, *CYP8B1*, *ACOX1* and *ACBP*) and four down-regulated genes (*PPARγ*, *FATCD36*, *UBC* and *PDK1*) between the control and rt-hLEP-injected fish (Table 3). The function of differentially expressed genes involved in fatty acid β-oxidation and oocytes maturation was summarized in Table 4.

#### 2.2.3. Differentially Expressed Genes Involved in Oocytes Maturation

To study if rt-hLEP induced oocytes maturation, we analysed differentially expressed genes involved in oocytes maturation. These differentially expressed genes were involved in a wide aspect of oocytes maturation, including progesterone-mediated oocyte maturation, oocyte meiosis signalling pathway, GnRH signalling pathway, and cell cycle signalling pathway (Table 3). The vast majority of genes involved in these pathways were up-regulated following rt-hLEP injection, such as *MAPK*, *RINGO*, *Cdc2*, *MEK1*, *IGF-1R*, *APC/C*, *Cdk2*, *GnRHR*, *STAG3*, *SMC1*, *FSHβ* and *C-My*c (Table 3; Figure 5C,D).

#### 2.2.4. Differentially Expressed Genes Involved in Other Pathways

To get a more comprehensive description of differentially expressed genes in present study, we also payed attention to other key pathways, except for those mentioned above. Analysis of these genes which were differentially expressed between control and rt-hLEP injection groups, revealed the signal transduction pathways involved, including mTOR signalling pathway, notch signalling pathway and calcium signalling. In addition, expression of genes involved in digestion and metabolism were significantly different between the control and rt-hLEP-injected fish, including fat digestion and absorption, and protein digestion and absorption (Supplementary Table S3).

#### 2.2.5. Validation of Differential Gene Expression by qPCR

The expression levels of 22 genes (18 up-regulated and four down-regulated genes) from RNA-seq were validated by qPCR analysis. The results showed that 20 genes exhibited a concordant direction both in RNA-seq and qPCR analysis except Echs1 (enoyl-CoA hydratases 1) and PKC (protein kinase C) genes (Supplementary Figure S3). The correlation coefficient between RNA-seq and qPCR results was 0.894 (*p* < 0.001).

#### 2.2.6. rt-hLEP Injection Activated Ovarian CPT-1 Activity 

**Figure 6:**
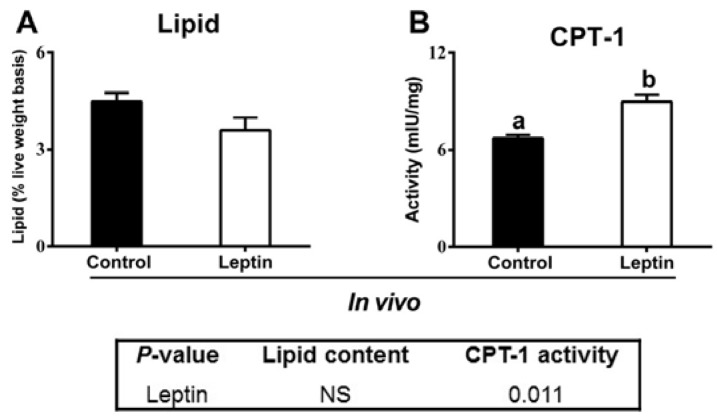
Effect of rt-hLEP on lipid content (**A**) and CPT-1 activity (**B**) in ovary of yellow catfish in vivo. Values are expressed as mean ± SEM (*n* = 3 replicate tanks, seven fish for ovarian lipid contents and four fish CPT-1 activity were sampled for each tank). Different letters indicated significant differences among groups (*p* < 0.05).

As a rate-limiting enzyme of fatty acid oxidation, the activity of CPT-1 also was measured. As shown in Figure 6, rt-hLEP injection significantly increased ovarian CPT-1 activity in yellow catfish. No significant differences were observed in ovarian lipid content between rt-hLEP-infected *P. fulvidraco* and control.

### 2.3. Leptin Induced Oocytes Maturation via Activation of JAK-STAT Pathway

**Figure 7:**
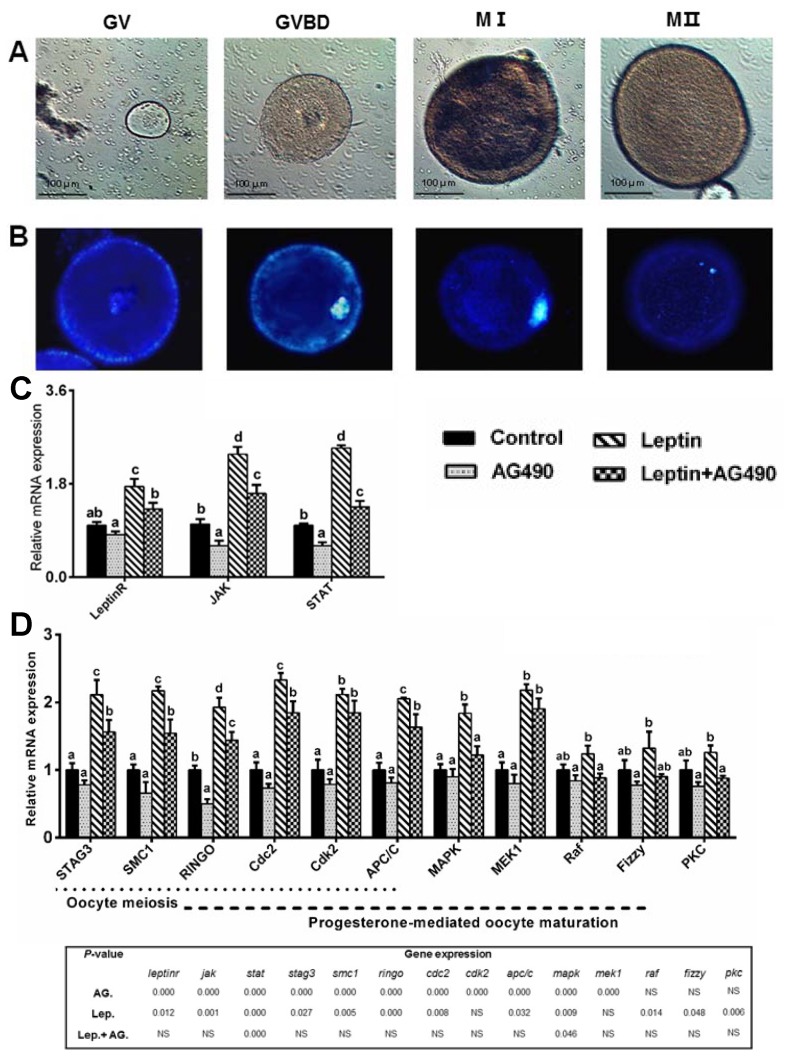
Leptin mediates oocytes maturation competence via activation of JAK-STAT pathway. (**A**) Morphology of yellow catfish oocytes at GV, GVBD, MI and MII phase, respectively (original magnification × 200, bars 100 μm). (**B**) Nuclear status of yellow catfish oocytes at GV, GVBD, MI and MII phase, respectively (original magnification × 400). Stained with Hoechst 33342. (**C**,**D**) Effect of AG490 on mRNA expression of genes involved in JAK-STAT pathway and oocytes maturation following rt-hLEP treatment in vitro. mRNA expression values were normalized to β-actin and GAPDH expressed as a ratio of the control (control = 1). Values are shown as mean ± SEM (*n* = 4 independent biological experiments). Different letters indicated significant differences among groups (*p* < 0.05).

Since leptin is considered to exert its biological actions primarily through JAK-STAT signaling pathway, using the primary oocytes of *P. fulvidraco*, specific inhibitors AG490 (JAK-STAT inhibitor) was used to explore the involvement of JAK-STAT pathway activation in leptin-mediated oocytes maturation in *P. fulvidraco*. First, a total of 119, 116, and 117 oocytes of control, leptin and leptin+AG490 groups were cultured and evaluated for nuclear maturation (Table 5). As shown in Table 5, the percentage of leptin-incubated oocytes arrested at GV stage was significantly lower than those of control groups (*p* < 0.05). The rate of meiotic resumption (GVBD-MII) of leptin-incubated oocytes was significantly higher than those of control groups (*p* < 0.05). Compared to the control, leptin significantly increased oocytes diameter in vitro (Table 5). Compared to individual leptin-treated group, leptin and AG490 co-incubation significantly increased the percentage of GV-stage oocytes, but reduced the rate of meiotic resumption (*p* < 0.05). Next, we investigate effect of AG490 and leptin treatment on mRNA expression of genes involved in JAK-STAT pathway and oocytes maturation of yellow catfish. Compared to the control, leptin significantly increased the mRNA levels of leptin receptor (LepR), JAK, STAT, cohesin complex subunit SA-3 (STAG3), structural maintenance of chromosome 1 (SMC1), RINGO, cyclin-dependent kinase 1 (Cdc2), cyclin-dependent kinase 2 (Cdk2), anaphase-promoting complex subunit 1 (APC/C), MAPK and mitogen-activated protein kinase 1 (MEK1) (Figure 7C,D); leptin and AG490 co-incubation significantly reduced the leptin-induced up-regulation of *LepR*, *JAK*, *STAT*, *STAG3*, *SMC1*, *RINGO*, *Cdc2*, *APC/C*, *Raf* and *MAPK* mRNA levels.

### 2.4. Fatty Acid β-Oxidation Is Essential for Leptin-Mediated Oocytes Maturation

**Figure 8:**
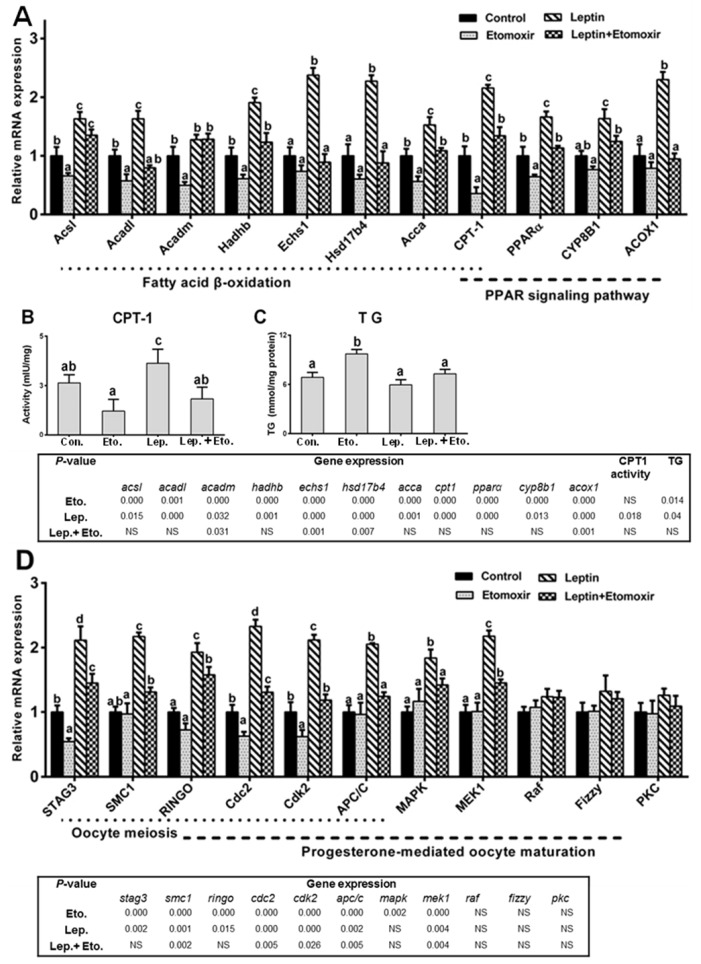
Fatty acid β-oxidation is essential for leptin-mediated oocytes maturation competence. (**A**) Effect of etomoxir on mRNA expression of genes involved in fatty acid β-oxidation in oocytes from yellow catfish with rt-hLEP treatment in vitro. (**B**,**C**) Effect of etomoxir on CPT-1 activity and TG content in oocytes with rt-hLEP treatment in vitro, respectively. (**D**) Effect of etomoxir on mRNA expression of genes involved in oocytes maturation of yellow catfish with rt-hLEP treatment in vitro. mRNA expression values were normalized to β-actin and GAPDH expressed as a ratio of the control (control = 1). Values are shown as mean ± SEM (*n* = 4 independent biological experiments). Different letters indicated significant differences among groups (*p* < 0.05).

To determine the effect of inhibiting β-oxidation on leptin-mediated oocytes maturation competence in *P. fulvidraco*, oocytes was treated with rt-hLEP with or without specific fatty acid β-oxidation inhibitors (etomoxir) for 48 h. As shown in Figure 8A–C, etomoxir was effective at inhibiting fatty acid β-oxidation in oocytes in vitro. Compared to the control, etomoxir decreased the mRNA levels of *Acsl*, *Acadl*, *Acadm*, *Acca*, *CPT-1* and *PPARα.* Similarly, compared to individual leptin-treated group, leptin and etomoxir co-incubation significantly reduced the mRNA levels of all the eleven tested genes involved in fatty acid β-oxidation, except for *Acsl* and *Acadm*. The inhibition by etomoxir was further confirmed by CPT-1 activity and TG content in oocytes in vitro.

Next, we investigated whether etomoxir-induced inhibition of fatty acid β-oxidation had an effect on leptin-induced oocytes maturation competence. Etomoxir was effective at inhibiting oocytes maturation in vitro. First, etomoxir significantly attenuated the leptin-induced elevation of maturation rate and diameter of oocytes (Table 5). Second, etomoxir tended to reduce the leptin-induced elevation of the mRNA levels of *STAG3*, *SMC1*, *RINGO*, *Cdc2*, *Cdk2*, *APC/C*, *MAPK* and *MEK1* (Figure 8D). In addition, effects of etomoxir on the mRNA levels of leptin and leptin receptor (LepR) in oocytes from yellow catfish with rt-hLEP treatment were shown in Supplementary Figure S4. Compared to individual leptin-treated group, leptin and etomoxir co-incubation significantly reduced the LepR mRNA levels.

### 2.5. Fatty Acid β-Oxidation up-Regulation Improved Leptin-Mediated Oocytes Maturation

**Figure 9:**
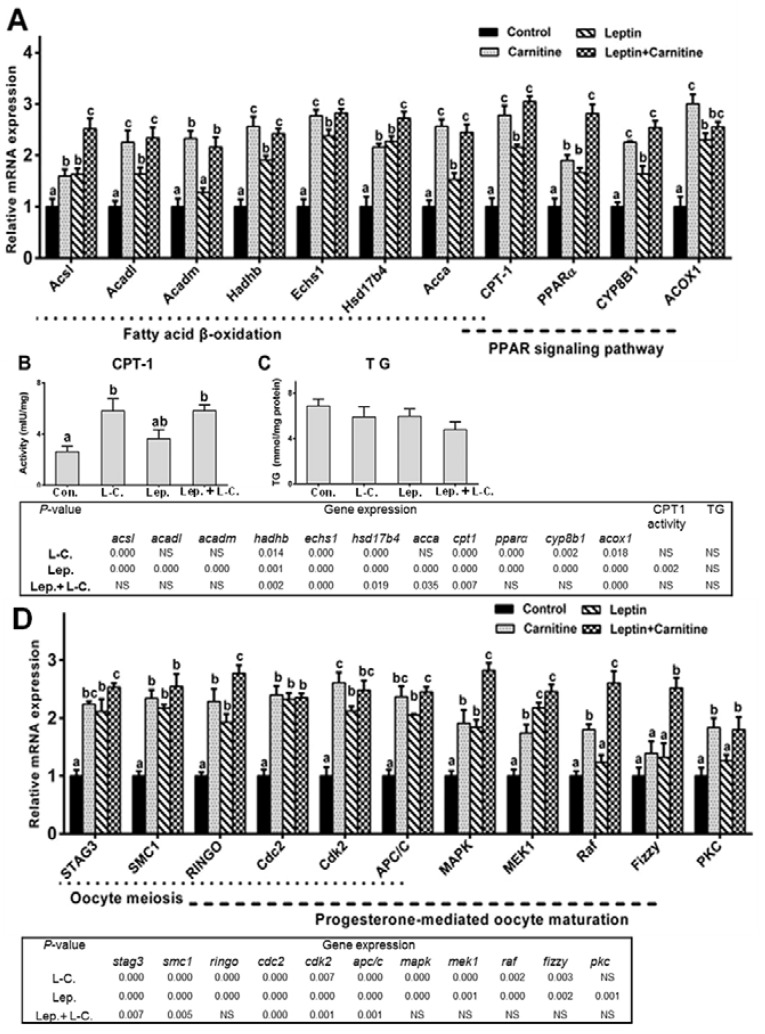
l-carnitine significantly up-regulates fatty acid β-oxidation in the oocytes and significantly improves leptin-mediated oocyte maturation competence in vitro. (**A**) Effect of l-carnitine on mRNA expression of genes involved in fatty acid β-oxidation in oocytes from yellow catfish with rt-hLEP treatment in vitro. (**B**,**C**) Effect of l-carnitine on CPT-1 activity and TG content in oocytes with rt-hLEP treatment in vitro. (**D**) Effect of l-carnitine on mRNA expression of genes involved in oocytes maturation with rt-hLEP treatment in vitro. mRNA expression values were normalized to β-actin and GAPDH expressed as a ratio of the control (control = 1). Values are shown as mean ± SEM (*n* = 4 independent biological experiments). Different letters indicated significant differences among groups (*p* < 0.05).

After we had determined that inhibition of fatty acid β-oxidation had detrimental effects on leptin-mediated oocytes maturation capacity in vitro, we next sought to investigate whether up-regulation of this metabolic pathway by supplementation of l-carnitine could improve leptin-mediated oocytes maturation potential. As shown in Figure 9A–C, l-carnitine supplementation alone caused a significant increase in CPT-1 activity and the mRNA levels of all the eleven tested genes involved in β-oxidation. Similarly, supplementation of both leptin and l-carnitine led to a significant up-regulation of the mRNA levels of all the eleven tested genes involved in β-oxidation, except for *ACOX1*, and CPT-1 activity when compared to individual leptin incubation.

After we had established that l-carnitine could significantly increase β-oxidation in oocytes, we next assessed whether l-carnitine had an effect on leptin-mediated oocytes maturation in vitro. First, leptin and l-carnitine co-incubation significantly increased the maturation rate and diameter of oocytes (Table 5). Second, single l-carnitine supplementation significantly increased the expression of all the eleven tested genes involved in oocytes maturation (Figure 9D). Similarly, compared to individual leptin supplementation, leptin and l-carnitine co-incubation significantly up-regulated the mRNA levels of RINGO, MAPK, MEK1, Raf and Fizzy. In addition, as shown in Supplementary Figure S4, compared to the control, l-carnitine increased the *LepR* gene expression levels. Similarly, compared to individual leptin-treated group, leptin and l-carnitine co-incubation significantly up-regulated the LepR mRNA levels.

## 3. Discussion

In spite of the plethora of investigations devoted to study leptin function, few studies have considered the molecular mechanism of leptin in fatty acid β-oxidation and oocytes maturation in fish. In this study, we investigated the differentially expressed genes involved in fatty acid β-oxidation and oocytes maturation in ovary of *P. fulvidraco* following rt-hLEP injection by RNA-seq technology. The presented study, for the first time, provided evidence that leptin was a very important factor that links fatty acid β-oxidation with oocytes maturation and leptin mediated oocytes maturation competence by activation of JAK-STAT pathway in fish. Furthermore, using primary culture of oocyte cells, we also revealed that up-regulation of β-oxidation could improve leptin-mediated oocytes maturation in fish.

In the present study, transcriptome analyses showed that rt-hLEP injection led significant changes in the expression of genes involved in fatty acid β-oxidation. CPT-1 is considered to be the main regulatory enzyme in long-chain fatty acid β-oxidation, catalysing the conversion of fatty acid-CoAs into fatty acid-carnitine for entry into the mitochondrial matrix [26]. Our study indicated that rt-hLEP injection increased the activity and expression of CPT-1. Similarly, rt-hLEP incubation significantly increased the activity and expression of CPT-1 in ovarian follicle cells from *P. fulvidraco* [13]. Also, Londraville and Duvall [27] reported that CPT-1 activity was higher in green sunfish *Lepomis cyanellus* following leptin treatment. Except for CPT-1, other genes (such as *Acca*, *Acsl*, *Hadhb*, and *Echsl*), which played crucial roles in β-oxidation [24], also were up-regulated by rt-hLEP injection in our study. The changes in the CPT-1 activity and expression of genes involved in β-oxidation suggested that rt-hLEP injection activated β-oxidation pathways in ovary of *P. fulvidraco*. Similarly, Yamagishi et al. [28] indicated that leptin incubation increased fatty acid β-oxidation by stimulating the activity of CPT-1 in bovine aortic endothelial cells in vitro. Minokoshi et al. [29] pointed out that leptin activated fatty-acid oxidation in red muscle of mice in vivo. In addition, several transcription factors (such as *PPARα* and *PPARγ*) play an intermediary role in β-oxidation, by orchestrating the transcription of the genes encoding enzymes involved in these pathways [30]. In the present study, following rt-hLEP infection, some differentially expressed genes were associated with “PPAR signaling pathway”, including PPARα, PPARγ, CYP8B1, ACOX1, ACBP, FATCD36, UBC and PDK1, suggesting the leptin-induced activation of fatty acid β-oxidation through, at least in part, PPAR signaling pathway.

Leptin has been shown to exert positive effects during the maturation of oocytes in mammals [1]. Direct effects of leptin on regulation of ovarian steroidogenesis have also been investigated both in vivo and in vitro [18]. In mammals, in vitro studies have shown a direct action of leptin at the hypothalamic–pituitary level; leptin induces LHRH release by hypothalamic explants and FSH and/or LH releases by pituitaries [19,20]. In fish, the present study indicated that certain genes involved in pathways for oocytes maturation, such as progesterone-mediated oocyte maturation, oocyte meiosis signalling pathway, GnRH signalling pathway, and cell cycle signalling pathway, were differentially regulated. Furthermore, in the current study, leptin incubation significantly increased the maturation rate and diameter of oocytes. Studies have shown the oocyte diameter related maturational competence in vitro [31]. Thus, the present results suggested that leptin could activate oocytes maturation pathways and improve oocytes maturation competence. Similarly, mammalian leptin administration led to increases in LH and somatolactin in sea bass [21]. Human recombinant leptin caused increased release of FSH and LH from the pituitary of female trout [22]. In addition, Paula-Lopes et al. [1] shown that leptin enhanced both oocyte maturation and differentially regulated gene expression in bovine oocytes and cumulus cells. Zhang et al. [32] reported that leptin enhanced oocyte nuclear and cytoplasmic maturation in porcine oocytes. On the other hand, the physiological actions of leptin are mediated by membrane-associated leptin receptor, which signals via common key intracellular signaling pathways [33,34]. In the current study, transcriptome analyses showed that many pathways for signal transduction were activated by rt-hLEP injection, including JAK-STAT signaling pathway, MAPK signaling pathway, and AMPK signaling pathway. Previous studies have reported that JAK/STAT, MAPK and AMPK pathways were involved in leptin’s biological functions [33,35,36]. The JAK-STAT pathway is thought a prime candidate to transmit the signal generated by leptin [23]. Therefore, to underpin efforts to further understand the underlying processes involved in leptin-induced oocyte maturation, we examined the potential role of JAK-STAT pathway in leptin-induced oocyte maturation. Our present study found that the specific JAK-STAT pathway inhibitor, AG490, significantly attenuated the leptin-induced elevation of maturation rate and diameter of oocytes, and markedly blocked the leptin-induced upregulation of gene expression involved in oocytes maturation, which imply JAK-STAT involved the leptin-induced oocytes maturation in *P. fulvidraco*. Similarly, Arias-Álvarez et al. [35] found that leptin influenced the maturation of cumulus–oocyte complexes from rabbit through JAK2/STAT3 in vitro.

The present study found the fatty acid β-oxidation pathway has an important role in establishing leptin-mediated oocytes maturation competence. Etomoxir resulted in the down-regulation of certain genes involved in pathways for fatty acid β-oxidation and CPT-1 activity, and the up-regulation of TG in vitro, indicating the inhibition of fatty acid β-oxidation with etomoxir in vitro. Similarly, previous study has also reported that etomoxir significantly decrease β-oxidation rates and CPT-1 activity in vitro [7,37]. Furthermore, the present study found the fatty acid β-oxidation pathway had an important role in establishing leptin-mediated oocytes maturation competence. First, the inhibition of fatty acid β-oxidation with etomoxir in vitro resulted in the down-regulation of certain genes involved in pathways for oocytes maturation. Second, blocking of fatty acid β-oxidation inhibited the maturation and reduced diameter of oocytes, indicating that fatty acid β-oxidation was involved in oocytes maturation. Similarly, Downs et al. [5] pointed out that β-oxidation was involved in oocyte meiotic resumption and etomoxir treatment was associated with significant impairment in GVBD. Also, Dunning et al. [7] indicated the inhibition of β-oxidation during oocyte maturation resulted in the reduction of oocytes developmental competence. In addition, the present study indicated that inhibition of β-oxidation with etomoxir significantly repressed the competence of leptin-mediated oocytes maturation, as evidenced by the fact that treatment of oocytes with etomoxir attenuated the leptin-induced increase of maturation rate, diameter of oocytes and gene expression involved in oocytes maturation, which further demonstrated that fatty acid β-oxidation was essential in leptin-mediated oocytes maturation.

On the other hand, l-carnitine supplementation significantly up-regulated the mRNA expression of certain genes involved in pathways for fatty acid β-oxidation and CPT-1 activity, and down-regulated TG in the oocytes, suggesting the up-regulation of β-oxidation in vitro by l-carnitine supplementation. Similarly, l-carnitine supplementation had been shown the up-regulation of β-oxidation and CPT-1 activity in mammal in vitro [7,38]. Moreover, the present study indicated that the up-regulation of β-oxidation in vitro by l-carnitine supplementation increased leptin-mediated oocytes maturation competence, as manifested by a significant improvement in maturation rate and diameter of oocytes, and also the significant up-regulation of genes involved in pathways for oocytes maturation. In vitro studies have shown that l-carnitine decreased oocyte cytoskeletal damage and have beneficial effects on murine embryo development in vitro [39,40]. Dunning et al. [7] found that l-carnitine resulted in significant up-regulation of the β-oxidation pathway, which suggested that the increase in oocyte developmental potential after l-carnitine inclusion may be the result of increased β-oxidation levels. Our results suggested l-carnitine supplementation could improve leptin-mediated oocytes maturation competence in vitro through increasing oocyte β-oxidation levels, which is closely associated with oocyte quality [7,41]. It should be pointed out that recombinant human LEP was used in the present study. Several studies pointed out that homologous leptin were probably better for discriminating the potential scope of physiological actions of leptin in fish while heterologous hormone might be useful in some instances [42,43].

In summary, the present study provided the ovarian transcriptome information of *P. fulvidraco* by RNA-seq technology, and clearly indicated that leptin is a very important factor that links fatty acid β-oxidation with oocytes maturation. Leptin induced oocytes maturation by activation of JAK-STAT pathway. Furthermore, the present study also revealed the important role of fatty acid β-oxidation in establishing leptin-mediated oocytes maturation competence, and promoting β-oxidation can improve leptin-mediated oocytes maturation outcomes in fish, which may be developed to improve oocytes developmental potential and alleviate sub-fertility in fish.

## 4. Materials and Methods

### 4.1. Reagents

HPLC-purified rt-hLEP, AG490, (R)-(+)-etomoxir, l-carnitine, and other reagents were purchased from Sigma-Aldrich Chemical Co (St. Louis, MO, USA). Mammalian-derived leptin orthologs were used in the present study since studies have indicated its role in *P. fulvidraco* [13,44].

### 4.2. Drug Treatment

rt-hLEP were dissolved in phosphate buffered saline (PBS) for in vivo experiment and dissolved in dimethyl sulfoxide (DMSO) for in vitro experiment. (R)-(+)-Etomoxir and l-carnitine were dissolved in DMSO. The maximal DMSO concentration applied to cells did not exceed 0.1%, and had no discernible effect on cell viability and other biological parameters. The concentrations of rt-hLEP (1.5 µg/g in vivo or 500 ng/mL in vitro) were selected according to our preliminary experiment and consistent with that used in our recent in vivo and in vitro studies in *P. fulvidraco* [13,43]. The dose of AG490 was selected according to our previous studies and preliminary experiment in yellow catfish [44]. The dose of (R)-(+)-etomoxir (100 µM) and l-carnitine (5 mM) was based upon our preliminary experiments and reports on mammals in vitro [7].

### 4.3. Experimental Treatments

Three experiments were conducted. In Experiment 1, *P. fulvidraco* were intraperitoneally injected with 1.5 µg rt-hLEP/g body weight (BW), and sampling occurred at 48 h after injection for ovarian transcriptomic analysis. In experiment 2, primary oocytes of *P. fulvidraco* were isolated, pre-treated with AG490 (an inhibitor for JAK-STAT pathway) and then with rt-hLEP treatment for 48 h. In Experiment 3, oocytes were treated with (R)-(+)-etomoxir (an inhibitor of β-oxidation) or l-carnitine (an enhancer of β-oxidation) with rt-hLEP treatment for 48 h. We ensured that the experiments were performed in accordance with the experimental protocols of Huazhong Agricultural University (HZAU) and approved by the ethics committee of HZAU (identification code: Fish-2015-0319, Date: 19 March 2015).

#### 4.3.1. Experiment 1: In Vivo Study: Investigating the Role and Molecular Mechanism of Leptin Underlying Fatty Acid β-Oxidation and Oocytes Maturation

To obtain mature oocytes, yellow catfish were collected from a local fish pond (Wuhan, China) in early May, and then transferred to indoor fiberglass tanks (400-L in water volume) for acclimation. Yellow catfish collected just prior to the spawning season generally contain oocytes in which the germinal vesicle is located approximately midway between the oocytes center and surface [45,46]. During the acclimation period, the fish were fed to apparent satiation twice daily with a commercial pellet diet (lipid and protein contents of 9.4% and 42.6% on a dry matter basis, respectively). At the beginning of the trial, 90 uniform-sized fish (mean weight: 26.36 ± 1.89 g, mean ± SEM) were stocked in 6 fiberglass tanks, with 15 fish in each tank. Then they were anesthetized with tricaine methanesulfonate (MS-222) at 100 mg/L and intraperitoneally injected with 1 μL/g fish BW of PBS (control) or with 1.5 μg rt-hLEP/g BW. Each treatment had three replicate tanks. After injection, fish were returned to their corresponding tanks.

Sampling occurred at 48 h after injection. Fish from each tank were euthanized (MS-222 at 100 mg/L). Eight fish per tank were randomly selected and dissected on ice. Then, their ovaries were removed immediately and stored at −80 °C for subsequent analysis (for each tank, four fish for RNA-Seq analysis and four fish for qPCR analysis). The ovaries from other seven fish were kept at −80 °C for determination of the lipid contents.

#### 4.3.2. Experiment 2: In Vitro Study: Investigating whether Leptin Induced Oocytes Maturation and Its Relationship with JAK-STAT Pathway

The same batch *P. fulvidraco* as Experiment 1 with similar body weight was used in Experiment 2. Oocytes were isolated from yellow catfish according to the published protocols [21] with slight modification. Briefly, yellow catfish were euthanized, and the ovaries were removed and held in ice-cooled Cortland’s salt solution [47]. Ovaries were cut into groups of follicles and immediately placed into individual incubation flask. Each flask contained 30 oocytes and these oocytes came from the same yellow catfish. Prior to incubation, the germinal vesicles of oocytes were located halfway between the centre and the oocyte periphery. During the intrafollicular oocytes, migration had already begun in some germinal vesicle prior to incubation. However, the germinal vesicles were still far from the oocytes surface. Cortland’s salt solution was used as the incubation medium. No antibiotics were added. 5 mL of medium were used for incubations. All incubations were carried out in 25 mL Erlenmeyer flasks.

To explore the role of JAK-STAT pathway mediating leptin-induced oocytes maturation, primary *P. fulvidraco* oocytes were treated with 20 µM AG490, JAK-STAT specific inhibitor. For the experiment, four groups were designed as follows: control (containing 0.1% DMSO), AG490 (20 μM), leptin (500 ng/mL), and leptin (500 ng/mL) + AG490 (20 μM), respectively. Each treatment was performed in octuplicate. Incubations were carried out at 18 °C for 48 h [48]. The half of oocytes were examined under a dissecting microscope for morphological assessment, and then stained with Hoechst 33342 for assessment of nuclear status as previously described [49]. They were examined using a fluorescence microscope with a 355-nm excitation wave length. The meiotic stages of in vitro matured oocytes were classified as previously described [50]: germinal vesicle (GV), germinal vesicle breakdown (GVBD), metaphase I (MI), and metaphase II (MII). The remaining oocytes were used for qPCR analysis.

#### 4.3.3. Experiment 3: In Vitro Study: Investigating the Potential Role of Fatty Acid β-Oxidation in Leptin-Mediated Oocyte Maturation

Using the primary oocytes of yellow catfish, specific inhibitors (Etomoxir) and activator (l-carnitine) of fatty acid β-oxidation were used to investigate the potential role of fatty acid β-oxidation in leptin-mediated oocyte maturation. Oocytes of *P. fulvidraco* were isolated and incubated according to the methods described above. (R)-(+)-Etomoxir is a well-characterized and nonreversible inhibitor of fatty acid β-oxidation [51]. To determine the effect of fatty acid β-oxidation inhibition on oocytes maturation, oocytes were treated with 100 µM etomoxir. Meanwhile, fatty acid β-oxidation was shown to occur basally in the absence of exogenous l-carnitine in vitro [7]. Thus, to investigate whether up-regulation of fatty acid β-oxidation could improve oocytes maturation, oocytes were treated with 5 mM l-carnitine. For the experiment, seven groups were designed as follows: control (0.1% DMSO), (R)-(+)-etomoxir (100 µM), l-carnitine (5 mM), leptin (500 ng/mL) and leptin (500 ng/mL) plus (R)-(+)-etomoxir (100 µM), leptin (500 ng/mL) plus l-carnitine (5 mM), respectively. The dose of (R)-(+)-etomoxir (100 µM) and l-carnitine (5 mM) was based upon our preliminary experiments and reports on mammals in vitro [7]. The etomoxir and l-carnitine were added 2 h prior to the addition of leptin. Each treatment was performed in octuplicate. Incubations were carried out at 18 °C for 48 h. The half of oocytes were examined under a dissecting microscope for morphological assessment, and then stained with Hoechst 33342 for assessment of nuclear status. The remaining half of oocytes were used for qPCR analysis.

### 4.4. Sample Analysis

#### 4.4.1. Total RNA Extraction, cDNA Library Preparation and Illumina Sequencing

Total RNA was isolated from each sample using Trizol reagent (Invitrogen, Carlsbad, CA, USA) according to the manufacturer’s protocol. All the samples were standardized to 500 ng/µL, and equal volumes of total RNA from twelve individuals (four fish each tank, and three tanks per group) in the same group were combined into one pool for transcriptome analysis, based on the published protocols [52,53].

The library construction and sequencing were performed by Beijing Genomics Institute (Shenzhen, China). The library was sequenced using Illumina HiSeq TM 2000 sequencing platform (San Diego, CA, USA).

#### 4.4.2. De Novo Assembly and Function Annotation

Transcriptome *de novo* assembly was carried out with Trinity paired-end assembly method (version r2013-02-25, Available online: http://trinityrnaseq.sourceforge.net/)53. All-Unigenes were submitted to databases for homolog and annotation comparison by BLASTx algorithm (*e*-value <10^−5^), including NR (non-redundant protein sequence), non-redundant nucleotide (Nt), Swiss-Prot, Kyoto Encyclopedia of Genes and Genomes (KEGG), and Clusters of Orthologous Groups (COG). Functional annotation by Gene Ontology terms (GO; Available online: http://www.geneontology.org) was accomplished with Blast2GO software [54]. After obtaining GO annotation for each unigene, WEGO software [55] was used to perform GO functional classification for All-Unigenes.

#### 4.4.3. Identification of Differentially Expressed Genes

Gene expression levels were calculated using the Reads Per kb per Million reads (RPKM) method [56]. A rigorous algorithm was developed to identify differentially expressed genes between the two treatments (control and leptin treatment) based on previously described methods [57]. The false discovery rate (FDR) was controlled to determine differentially expressed genes [58]. In this study, FDR ≤ 0.001 and the absolute value of log_2_ ratio ≥ 1 (fold-change ≥ 2) were used as the threshold to judge the significance of gene expression differences. For pathway and GO enrichment analysis, all differentially expressed genes were mapped to terms in GO and the KEGG database.

#### 4.4.4. Real-Time Quantitative PCR (qPCR) Validation

To validate the reliability of the Illumina analysis, twenty-two differentially-expressed genes that are mainly involved in pathways for signal transduction, fatty acid β-oxidation and oocytes maturation were selected for validation by qPCR analysis from twelve individuals (four fish each tank, and three tanks per group) described in our studies [43]. Primer were listed in Supplementary Table S1. A set of eight housekeeping genes (β-actin, GAPDH, EF1A, 18S rRNA, HPRT, B2M, TUBA and RPL17) were selected from the literature [59] and our transcriptome database in order to test their transcription stability. The relative expression levels were calculated using the 2^−ΔΔ*C*t^ method [60] when normalizing to the geometric mean of the best combination of two genes (β-actin and GAPDH, M = 0.28) as suggested by geNorm [61]. Prior to the analysis, experiments were performed to check the stability of housekeeping genes, from which β-actin and GAPDH showed the most stable level of expression under the experimental conditions.

#### 4.4.5. Determination of Lipid and Triglyceride (TG) Contents, and CPT-1 Activity

Ovarian lipid content was determined by the ether extraction [62]. TG was determined by glycerol-3-phosphate oxidase p-aminophenol (GPO-PAP) methods, using a commercial kit from Nanjing Jian Cheng Bio-engineering Institute, Nanjing, China. For the determination of CPT-1 activities, mitochondria were first isolated from the cells according to Suarez and Hochachka [63] with modification by Morash et al. [64]. CPT-1 activity was determined using the method of Bieber and Fiol [65], based on measurement of the initial CoA-SH formation by the 5,5′-dithio-bis-(2-nitrobenzoic acid) (DTNB) reaction from palmitoyl-CoA with l-carnitine at 412 nm.

### 4.5. Statistical Analysis

Statistical analysis was performed with SPSS 17.0 software (SPSS, Michigan Avenue, Chicago, IL, USA). Results were presented as mean ± standard error of means (SEM). Prior to statistical analysis, an arcsine transformation was used before processing percentage data. All data were tested for normality of distribution using the Kolmogorov–Smirnov test. The homogeneity of variances among the different treatments was tested using the Bartlett’s test. Then, they were subjected to one-way ANOVA and Tukey’s multiple range tests. The minimum significant level was set at 0.05.

## Figures and Tables

**Table 1:** Summary of output statistics by Illumina sequencing.

Samples	Total Clean Reads	Total Clean Nucleotides (nt)	Q20 Percentage	N Percentage	GC Percentage
Control	50,181,922	5,018,192,200	97.19%	0.00%	48.55%
Leptin injection	41,408,858	4,140,885,800	97.95%	0.00%	48.03%

**Table 2:** Statistics of assembly quality.

Parameters	Sample	Total Number	Total Length (nt)	Mean Length (nt)	N50 ^a^ (nt)
Contig	Control	76,153	39,303,110	516	1631
	Leptin injection	75,384	38,943,257	517	1650
Unigene	Control	45,002	46,286,299	1029	2217
	Leptin injection	44,503	45,719,364	1027	2232
All-Unigene		36,149	49,948,406	1382	2440

^a^ N50 = median length of all non-redundant sequences.

**Table 3:** Summary of differentially expressed genes involved in fatty acid β-oxidation and oocytes maturation in ovary of *P. fulvidraco* after rt-hLEP injection in vivo.

Pathways	Up-Regulated Genes	Down-RegulaTed Genes	Pathway ID (*p*-Value)
**Signal Transduction**			
JAK-STAT signaling pathway	*Leptin R*, *JAK*, *STAM*, *CI*S, *C-Myc*, *PI3K*	——	map04630 (0.000242)
MAPK signaling pathway	*MAPK, MEK1*, *MEKK*, *PP3C*, *HSP72*, *CASP*, *IL1*, *CrkII*, *C-Myc*	*Raf*, *PKC*	map04010 (0.000472)
AMPK signaling pathway	*LeptinR, CPT1, AMPK, PI3K, Acc, IGF-1R*	*PPARγ*	map04152 (0.00180)
**β-Oxidation**			
Fatty acid β-oxidation	*CPT1*, *Acsl*, *Acadl*, *Acadm*, *Hadhb*, *Echsl*, *Hsd17b4, Acca*	*Acads*	——
PPAR signaling pathway	*CPT1*, *PPARα*, *CYP8B1*, *ACOX1*, *ACBP*	*PPARγ*, *FATCD36*, *UBC*, *PDK1*	map03320 (0.00773)
**Oocytes Maturation**			
Progesterone-mediated oocyte maturation	*PI3K*, *MAPK, RINGO*, *Cdc2*, *MEK1*, *IGF-1R*, *APC/C*	*Raf*, *Fizzy*	map04914 (0.00309)
Oocyte meiosis signaling pathway	*RINGO*, *APC/C*, *Cdc2*, *Cdk2, STAG3*, *SMC1*	*C3H-4*	map04114 (0.0164)
GnRH signaling pathway	*GnRHR*, *MEKK*, *CREB*, *HB-EGF*, *FSHβ*	*Raf*, *PKC*	map04912 (0.0242)
Cell cycle signaling pathway	*APC/C*, *SMC1*, *Cdh1*, *Cdk2*, *C-Myc*	——	map04110 (0.000123)

**Table 4:** The function of differentially expressed genes involved in fatty acid β-oxidation and oocytes maturation.

DEGs	Functions
Leptin R	a receptor for the fat cell-specific hormone leptin
JAK	a family of intracellular tyrosine kinases that transduce cytokine-mediated signals via the JAK-STAT pathway
STAM (signal transducing adaptor molecule)	identified by the rapid tyrosine-phosphorylation of its product in response to cytokine stimulation
CIS (cytokine inducible SH2-containing protein)	cytokine-inducible negative regulators of cytokine signaling
C-Myc (proto-oncogene C-Myc)	a regulator gene that plays a role in cell cycle progression, apoptosis and cellular transformation
PI3K (phosphatidylinositol 3-kinase)	a family of enzymes involved in cellular functions such as cell growth, proliferation, differentiation, motility, survival and intracellular trafficking
MEK1 (mitogen-activated protein kinase kinase 1)	stimulates the enzymatic activity of MAP kinases upon activation by a wide variety of extra- and intracellular signals
MEKK (mitogen-activated protein kinase kinase kinase)	a serine/threonine kinase that occupies a pivotal role in a network of phosphorylating enzymes integrating cellular responses to a number of mitogenic and metabolic stimuli
PP3C (serine/threonine-protein phosphatase 2B catalytic subunit)	involved in a wide range of biologic activities, acting as a Ca^2+^-dependent modifier of phosphorylation status
HSP72 (heat shock 70 kDa protein 1/A)	a member of the heat shock protein 70 family which facilitates the proper folding of newly translated and misfolded proteins, as well as stabilize or degrade mutant proteins
CASP (caspase)	a family of protease enzymes playing essential roles in programmed cell death (including apoptosis, pyroptosis and necroptosis) and inflammation
IL1 (interleukin 1)	plays a central role in the regulation of immune and inflammatory responses to infections or sterile insults
CrkII (proto-oncogene C-crk)	a member of an adapter protein family that binds to several tyrosine-phosphorylated proteins and involved in several signaling pathways, recruiting cytoplasmic proteins in the vicinity of tyrosine kinase through SH2-phosphotyrosine interaction
Raf (B-Raf proto-oncogene serine/threonine-protein kinase)	part of the ERK1/2 pathway as a MAP kinase kinase kinase (MAPKKK) that functions downstream of the Ras subfamily of membrane associated GTPases
PKC (protein kinase C)	a family of protein kinase enzymes involved in controlling the function of other proteins through the phosphorylation of hydroxyl groups of serine and threonine amino acid residues on these proteins, or a member of this family
CPT (carnitine palmitoyltransferase)	a mitochondrial enzyme responsible for the formation of acyl carnitines by catalyzing the transfer of the acyl group of a long-chain fatty acyl-CoA from coenzyme A to l-carnitine
AMPK (5′-AMP-activated protein kinase)	an enzyme that plays a role in cellular energy homeostasis
Acc (acetyl-CoA carboxylase)	a biotin-dependent enzyme that catalyzes the irreversible carboxylation of acetyl-CoA to produce malonyl-CoA through its two catalytic activities, biotin carboxylase and carboxyltransferase
IGF-1R (insulin-like growth factor 1 receptor)	a transmembrane receptor that mediates the effects of IGF-1
PPAR (peroxisome proliferator-activated receptor)	a group of nuclear receptor proteins that function as transcription factors regulating the expression of genes involved in cellular differentiation, development, and metabolism (carbohydrate, lipid, protein), and tumorigenesis of higher organisms
Acsl (long-chain acyl-CoA synthetase)	converts free long-chain fatty acids into fatty acyl-CoA esters
Acadm (acyl-CoA dehydrogenase)	a class of enzymes that function to catalyze the initial step in each cycle of fatty acid β-oxidation in the mitochondria
Hadhb (hydroxyacyl-CoA dehydrogenase, β subunit)	functions in the mitochondrial matrix to catalyze the oxidation of straight-chain 3-hydroxyacyl-CoAs
Echs (enoyl-CoA hydratases)	an enzyme that hydrates the double bond between the second and third carbons on acyl-CoA
Hsd17b4 (hydroxyacysteroid 17-β dehydrogenase)	a group of alcohol oxidoreductases which catalyse the dehydrogenation of 17-hydroxysteroids in steroidogenesis
Acads (acyl-CoA dehydrogenase, short-chain)	an enzyme with systematic name short-chain acyl-CoA: electron-transfer flavoprotein 2,3-oxidoreductase
CYP8B1 (sterol 12-alpha-hydroxylase)	A member of the family of oxidoreductases, specifically those acting on paired donors, with O_2_ as oxidant and incorporation or reduction of oxygen
ACO (acyl-CoA oxidase)	A member of the family of oxidoreductases, specifically those acting on the CH-CH group of donor with oxygen as acceptor
ACBP (diazepam-binding inhibitor)	encodes diazepam binding inhibitor, a protein that is regulated by hormones and involved in lipid metabolism and the displacement of ß-carbolines and benzodiazepines, which modulate signal transduction at type A γ-aminobutyric acid receptors located in brain synapses
FATDC36 (CD36 antigen)	Leucocyte antigens on cell surfaces which recognizes oxidized low density lipoprotein, long chain fatty acids, anionic phospholipids, collagen types I, IV and V, thrombospondin and plasmodium falciparum infected erythrocytes
UBC (ubiquitin C)	plays a key role in maintaining cellular ubiquitin levels under stress conditions
PDK1 (3-phosphoinositide dependent protein kinase-1)	a master kinase crucial for the activation of AKT/PKB and many other AGC kinases including PKC, S6K, SGK
RINGO	a Cdc2 and Cdk2 activator, whose accumulation seems to be required for progesterone-induced oocyte maturation
Cdc (cyclin-dependent kinase; Cdk)	a family of protein kinases which are first discovered for their role in regulating the cell cycle
Fizzy (fizzy/cell division cycle 20 related 1)	Fizzy directly bind to anaphase-promoting complex and activate its cyclin ubiquitination activity
STAG3 (cohesin complex subunit SA-3)	a subunit of the cohesin complex which regulates the cohesion of sister chromatids during cell division
SMC1 (structural maintenance of chromosome 1)	A member of the family of proteins required for chromatid cohesion and DNA recombination during meiosis and mitosis
C3H-4 (CCCH zinc finger protein C3H-4)	encodes a CCCH-type zinc finger protein that is thought to prevent infection by retroviruses and may function to inhibit viral gene expression and induce an innate immunity to viral infection
GnRHR (gonadotropin-releasing hormone receptor)	a member of the seven-transmembrane, G-protein coupled receptor family and responsible for eliciting the actions of LHRH after its release from the hypothalamus
CREB (cyclic AMP-dependent transcription factor ATF-4)	a cellular transcription factor
HB-EGF (heparin-binding EGF-like growth factor)	play a role in wound healing, cardiac hypertrophy, and heart development and function
FSH (follicle stimulating hormone)	a glycoprotein polypeptide hormone which regulates the development, growth, pubertal maturation, and reproductive processes of the body
APC/C (anaphase-promoting complex subunit 1)	an E3 ubiquitin ligase that marks target cell cycle proteins for degradation by the 26S proteasome

**Table 5:** Mean (±SEM) nuclear status and diameter of yellow catfish oocytes after in vitro culture for 48 h in various media.

Treatment	No. Oocytes Cultured	Nuclear Status (%)					Mean Diamater of Oocytes (μm)
		GV	GVBD	M I	M II	GVBD-MII	
Control	119	20.8 ± 2.8 de	46.7 ± 3.0 c	28.3 ± 1.0 b	4.2 ± 1.6 a	79.2 ± 2.8 b	103.9 ± 2.8 b
AG490	120	11.7 ± 2.2 bc	44.2 ± 3.2 bc	35.0 ± 1.7 c	9.2 ± 2.5 abc	88.3 ± 2.2 cd	115.9 ± 2.3 cd
Etomoxir	119	34.2 ± 2.5 f	44.2 ± 2.8 bc	17.5 ± 1.6 a	3.3 ± 2.3 a	65.8 ± 2.5 a	89.3 ± 2.0 a
Carnitine	120	7.5 ± 2.1 ab	40.0 ± 3.0 abc	44.2 ± 3.2 d	8.3 ± 1.7 abc	92.5 ± 2.1 d	122.5 ± 2.5 df
Leptin	116	5.8 ± 1.6 ab	33.3 ± 1.4 a	40.8 ± 2.1 d	16.7 ± 4.1 c	94.2 ± 1.6 d	128.0 ± 1.6 f
Leptin + AG490	117	15.8 ± 1.6 cd	43.3 ± 3.0 bc	32.5 ± 1.6 bc	5.8 ± 3.7 ab	84.1 ± 1.6 bc	107.9 ± 3.8 bc
Leptin + Etomoxir	120	24.2 ± 1.6 e	40.8 ± 2.8 abc	29.7 ± 1.6 bc	5.8 ± 3.4 ab	75.8 ± 1.6 b	105.4 ± 3.0 b
Leptin + Carnitine	116	3.3 ± 1.4 a	35.8 ± 1.6 ab	44.2 ± 2.5 d	13.3 ± 1.4 bc	96.7 ± 1.4 d	125.9 ± 3.0 f

Within the same column, values without a common letter differed (*p* < 0.05).

## Availability of Data and Materials

The sequencing data in this study have been deposited in the Short Read Archive (SRA) at the National Centre for Biotechnology Information (NCBI) (https://www.ncbi.nlm.nih.gov/) under accession number: 352 SRX1080987.

## Supplementary Materials

The following are available online at http://www.mdpi.com/1422-0067/19/5/1457/s1.

## Abbreviations

BW	Body weight
COC	Cumulus-oocyte complex
COG	Clusters of Orthologous Groups
CPT	Carnitine palmitoyltransferase
DMSO	Dimethyl sulfoxide
FDR	False discovery rate
FSH	Follicle-stimulating hormone
GnRH	Gonadotropin releasing hormone
GO	Gene Ontology
GV	Germinal vesicle
GVBD	Germinal vesicle breakdown
JAK	Janus kinase
KEGG	Kyoto Encyclopedia of Genes and Genomes
LepR	Leptin receptor
LEP	Leptin
LH	Luteinizing hormone
MAPK	Mitogen-activated protein kinase
NCBI	National Center for Biotechnology Information
PBS	Phosphate buffered saline
PKC	Protein kinase C
PPAR	Peroxisome proliferator-activated receptor
qPCR	Real-time quantitative PCR
SRA	Short read archive
STAT	Signal transducer and activator of transcription
TG	Triglyceride
