# Supplementary material for: Fatty Acid β-Oxidation Is Essential in Leptin-Mediated Oocytes Maturation of Yellow Catfish Pelteobagrus fulvidraco
Source: Int J Mol Sci. 2018 May 14;19(5):1457. doi: 10.3390/ijms19051457 (PMC5983613; doi:10.3390/ijms19051457)

Supplementary Data

Supplementary Table 1 Primers used for qPCR analysis.

Supplementary Table 2 Summary of annotation results.

Supplementary Table 3 Summary of differentially expressed genes involved in other pathways in ovary of *P. fulvidraco* after rt-hLEP injection *in vivo.*

Supplementary Fig. 1 The length distribution of All-Unigene.

Supplementary Fig. 2 GO classification analysis of All-Unigene. GO functions are shown in *X* axis. The *right Y* axis shows the numbers of genes which have the GO function, and the *left Y* axis shows the percentage.

Supplementary Fig. 3 Comparison of relative expression levels between RNA-seq and qPCR results. The y-axis is the gene expressed fold change and the x-axis is the gene name.

Leptin R, leptin receptor; *C-Myc*, proto-oncogene c-myc*; PI3K,* phosphatidylinositol 3-kinase; *Acsl*, long-chain acyl-CoA synthetase; *CPT*, carnitine palmitoyltransferase; *Hadhb*, hydroxyacyl-CoA dehydrogenase, β subunit; *Echs*, enoyl-CoA hydratases; *Acsds*, acyl-CoA dehydrogenases; *Hsd17b4*, hydroxyacysteroid 17-*β* dehydrogenase; *Acc*, acetyl-CoA carboxylase; *CYP8B1*, sterol 12-alpha-hydroxylase; *ACO*, acyl-CoA oxidase; *FATDC36*, CD36 antigen; *MEK1*, mitogen-activated protein kinase kinase 1; *Raf*, B-Raf proto-oncogene serine/threonine-protein kinase; *PKC*, protein kinase C; *MAPK*, mitogen-activated protein kinase; *Cdc*, cyclin-dependent kinase; *APC*, anaphase-promoting complex; *STAG3*, cohesin complex subunit SA-3; *SMC1*, structural maintenance of chromosome 1; *Cdk2*, cyclin-dependent kinase 2.

Supplementary Fig. 4. Effects of etomoxir and carnitine on the mRNA levels of leptin and LepR in oocytes from yellow catfish with rt-hLEP treatment *in vitro*. mRNA expression values were normalized to β-actin and GAPDH expressed as a ratio of the control (control=1). Values are expressed as mean ± SEM (n= 4 independent biological experiments). Different letters

indicated significant differences among groups (P< 0.05).

Supplementary Table 1

Gene	Forward primer (5'-3')	Reverse primer (5'-3')
Leptin R	TACGTCCACCGTATCTGCAA	GCTGCTCAGACTCTCCTGCT
C-Myc	GATGAACGCGTGAAGTCTGA	ATATCCGAAAGCAGGGGACT
PI3K	TGCAGCCTTCAACAAAGATG	TCCCCAATTCCCAAAACATA
Acsl	ACACATGTTCGAGCGAGTTG	GGTGGGCCTCAGAGTCATAA
CPT-1	GTCAACAATGAGACGCGAGA	GCCATTTGGTACATGTGCTG
Hadhb	CTGGCCATAAAACACCGAGT	AGTGCATCCCAATCACCTTC
Echs1	TTTTTGGCACACTGGAACAA	TTCTGGCTGTCCAAACTGTG
PPARα	CGAGGATGGGATGCTGGTG	CGTCTGGGTGGTTCGTCTGC
Hsd17b4	TGGTTGATGAAACCACCAGA	TTGAATCCTCCCATTTCTGC
Acca	GCATTGCGTGAAGAGAACAA	TCAGCATGGTGACCAGAGAG
CYP8B1	GCTGGCTTATGGAGTTCTGG	GTCTGCTGCGATTCAGACAC
ACOX1	CCGGACATCTACGTCACCTT	TACGAGACGATGCCACTCAG
FATDC36	AGGTGCTCCTGTGCTCATTT	CGTCCAGGTAGGTGGAGTGT
MEK1	GTTGAGGGTCTCCATGCAAT	AGCGCATATTCAGGAGCATT
Raf	GGCCGTCTTTAACGGAGAG	CTCCTCGGGAATGTCGTG
PKC	CTTCAAGCAGCCCACATTCT	AAACAGCACACTTGGCACTG
MAPK	TGGTTTGCTGATGACACCAT	CGTTGGCTTGATATCCCATT
Cdc2	CCACTTCCTCCGACTCTCAG	GGGGTACTGCACTCCTGTGT
APC/C	CCTCAGCGCTGCTATTAACC	TTCACCAGAGGGTTGGACTC
STAG3	GCTCTGGGAGCAACACTTTC	TGTGGGGTGAGTGTGTGTTT
SMC1	GAGCAAATGAAGGCGAAAAG	GTCTTGGTCTGCTCGAGGTC
Cdk2	ACAAAATCACAGGCGAAACC	TGGGGTGATTGAGTTCCTTC
GAPDH	CATTACGAGGGCACGCTT	GGAGTTTCTGGACTTTAGGGAT
β-actin	ATCTTGCGTCTGGACTTGG	AGGCAGCGGTGTTCATTT

Leptin R, leptin receptor; *C-Myc*, proto-oncogene c-myc; *PI3K*, phosphatidylinositol 3-kinase;

Acsl, long-chain acyl-CoA synthetase; *CPT*, carnitine palmitoyltransferase; *Hadhb*, hydroxyacyl-CoA dehydrogenase, β subunit; *Echs*, enoyl-CoA hydratases; *PPAR*, Peroxisome proliferator-activated receptor; *Hsd17b4*, hydroxyacysteroid 17-*β* dehydrogenase; *Acc*, acetyl-CoA carboxylase; *CYP8B1*, sterol 12-alpha-hydroxylase; *ACO*, acyl-CoA oxidase; *FATDC36*, CD36 antigen; *MEK1*, mitogen-activated protein kinase kinase 1; *Raf*, B-Raf proto-oncogene serine/threonine-protein kinase; *PKC*, protein kinase C; *MAPK*, mitogen-activated protein kinase; *Cdc2*, cyclin-dependent kinase 1; *APC*, anaphase-promoting complex; *STAG3*, cohesin complex subunit SA-3; *SMC1*, structural maintenance of chromosome 1; *Cdk2*, cyclin-dependent kinase 2.

Supplementary Table 2

	Number of unigene hits	Percentage[a]
All-unigenes	36,149	—
All annotated unigenes	27,241	75.36%
Annotated to NR database	24,275	67.15%
Annotated to NT database	23,751	65.70%
Annotated to Swiss-Prot database	22,768	62.98%
Annotated to KEGG database	18,950	52.42%
Annotated to COG database	9,852	27.25%
Annotated to GO database	16,835	46.57%

[a] Proportion of the 36,149 assembled All-unigenes

Supplementary Table 3

Pathways	Up-regulated genes	Down-regulated genes	Pathway ID
Signal transduction			
mTOR signaling pathway	*STK11*, *RAPTOR*	*PDPK1*	map04150
Notch signaling pathway	*NOTCH1*, *PSEN1*	——	map04330
Calcium signaling	*SLC8A*, *F2R*, *EGFR*, *PHKG*, *PPP3C*, *MYLK*	*RYR1*	map04020
Digestion and metabolism			
Fat digestion and absorption	*FATP4*, *MGAT2*, *APOB*	*CD36*	map04975
Protein digestion and absorption	*COL1A*, *PAT1*, *SLC8A*, *CPB1*, *CTRB1*	——	map04974

STK11, serine/threonine-protein kinase 11; *RAPTOR*, regulatory associated protein of mTOR; *PDPK1*, 3-phosphoinositide dependent protein kinase-1; *PSEN1*, presenilin 1; *SLC8A*, solute carrier family 8; *F2R*, coagulation factor II (thrombin) receptor; *EGFR*, epidermal growth factor receptor; *PHKG*, phosphorylase kinase gamma subunit; *PPP3C*, serine/threonine-protein phosphatase 2B catalytic subunit; *MYLK*, myosin-light-chain kinase; *RYR1*, ryanodine receptor 1; *FATP4*, solute carrier family 27 member 4; *MGAT2*, 2-acylglycerol O-acyltransferase 2; *APOB*, apolipoprotein B; *CD36*, CD36 antigen; *COL1A*, collagen, type I, alpha; *PAT1*, solute carrier family 36 member 1; *CPB1*, carboxypeptidase B; *CTRB1*, chymotrypsinogen B1.

Supplementary Fig. 1

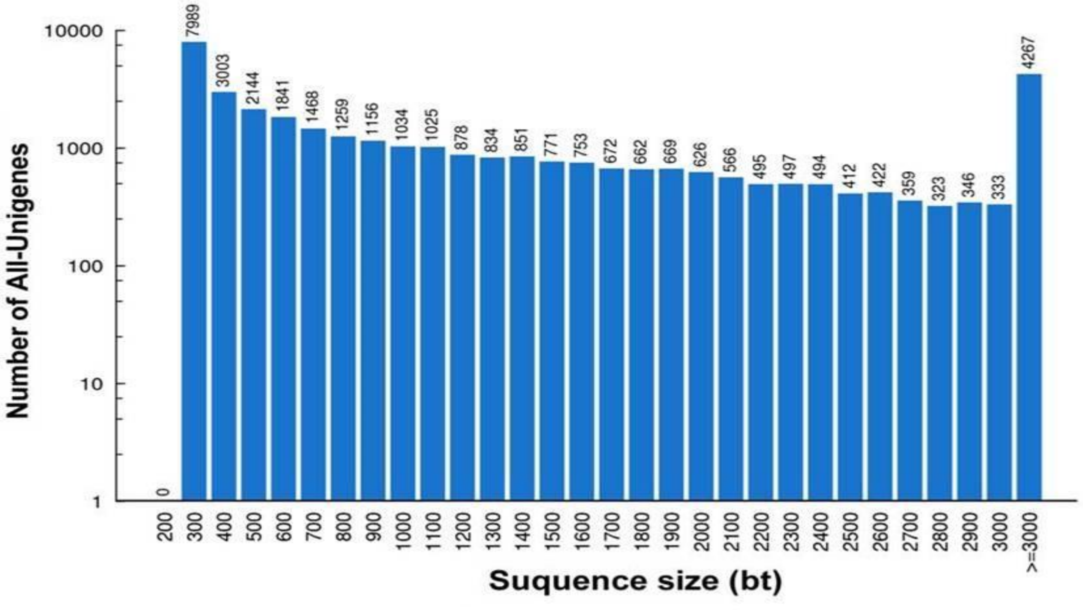

Supplementary Fig. 2

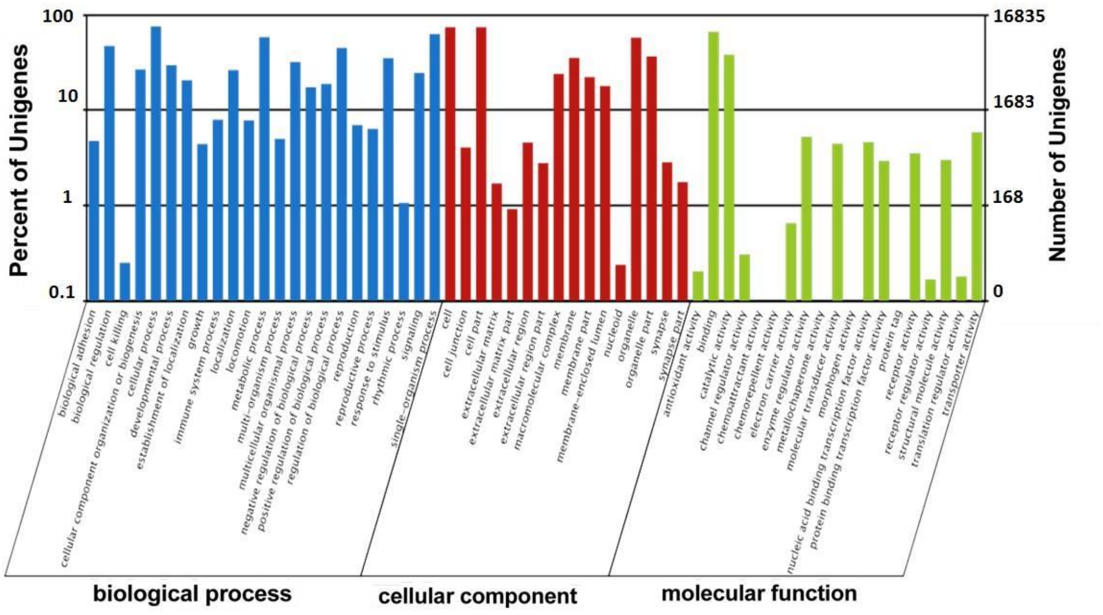

Supplementary Fig. 3

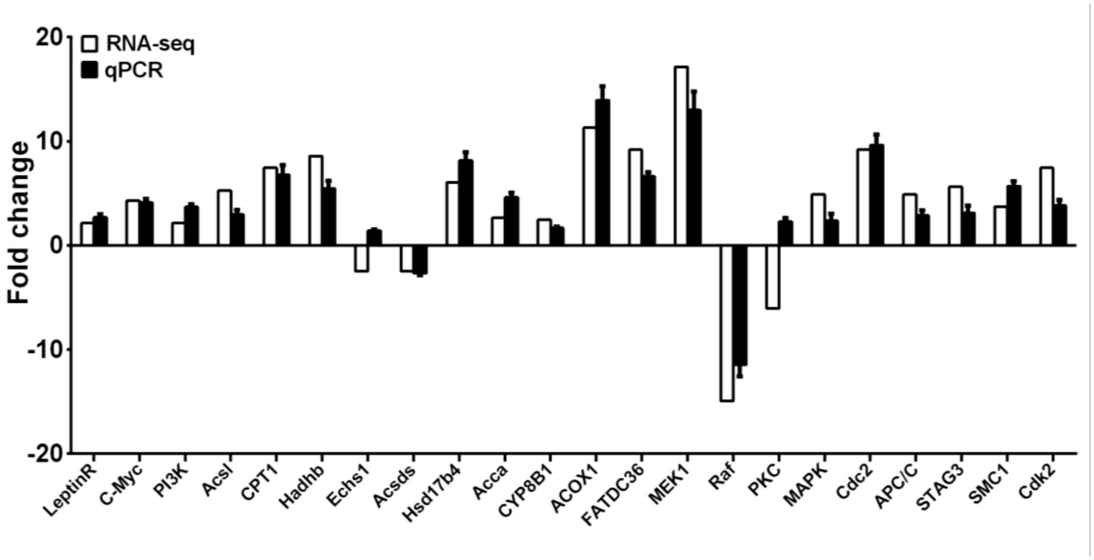

Supplementary Fig. 4.

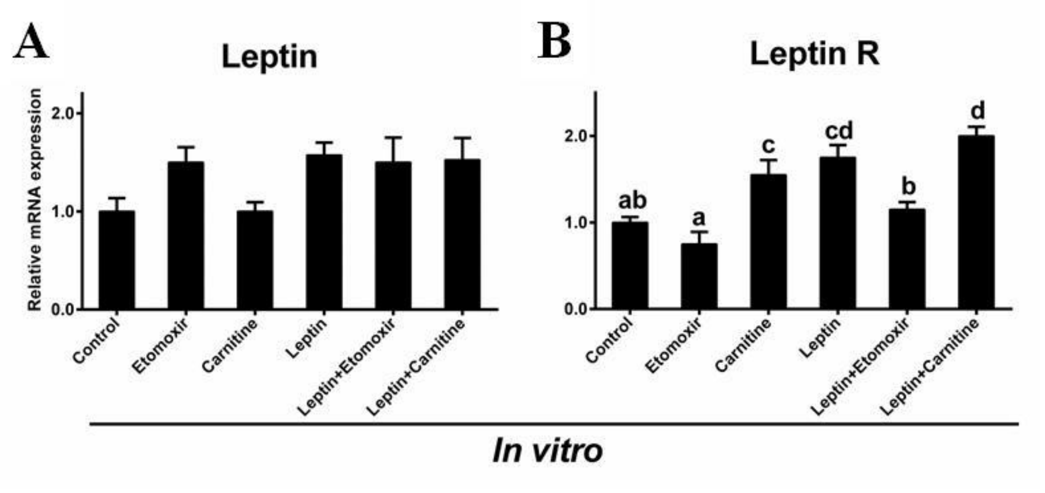

Supplement: Supplementary file 1 [file ijms-19-01457-s001.pdf]
